# The Role of Microglia and Astrocytes in Huntington’s Disease

**DOI:** 10.3389/fnmol.2019.00258

**Published:** 2019-10-25

**Authors:** Thulani H. Palpagama, Henry J. Waldvogel, Richard L. M. Faull, Andrea Kwakowsky

**Affiliations:** Centre for Brain Research, Department of Anatomy and Medical Imaging, Faculty of Medical and Health Sciences, University of Auckland, Auckland, New Zealand

**Keywords:** neuroinflammation, huntington’s disease, neurodegeneration, astrocytes, microglia

## Abstract

Huntington’s disease (HD) is an autosomal dominant neurodegenerative disease. HD patients present with movement disorders, behavioral and psychiatric symptoms and cognitive decline. This review summarizes the contribution of microglia and astrocytes to HD pathophysiology. Neuroinflammation in the HD brain is characterized by a reactive morphology in these glial cells. Microglia and astrocytes are critical in regulating neuronal activity and maintaining an optimal milieu for neuronal function. Previous studies provide evidence that activated microglia and reactive astrocytes contribute to HD pathology through transcriptional activation of pro-inflammatory genes to perpetuate a chronic inflammatory state. Reactive astrocytes also display functional changes in glutamate and ion homeostasis and energy metabolism. Astrocytic and microglial changes may further contribute to the neuronal death observed with the progression of HD. Importantly, the degree to which these neuroinflammatory changes are detrimental to neurons and contribute to the progression of HD pathology is not well understood. Furthermore, recent observations provide compelling evidence that activated microglia and astrocytes exert a variety of beneficial functions that are essential for limiting tissue damage and preserving neuronal function in the HD brain. Therefore, a better understanding of the neuroinflammatory environment in the brain in HD may lead to the development of targeted and innovative therapeutic opportunities.

## Introduction

Initially, neuroinflammation has been used to describe the infiltration of peripheral immune cells in the central nervous system (CNS), however, the term is currently also used in relation to neurodegenerative diseases. In these conditions such as Huntington’s disease (HD), Alzheimer’s disease (AD), Parkinson’s disease (PD) and Amyotrophic lateral sclerosis (ALS) neuroinflammation is characterized by a reactive morphology of glial cells, including both astrocytes and microglia, along with the presence of inflammatory mediators in the brain parenchyma (Ransohoff, 2016a; Masgrau et al., 2017). Previous studies have identified changes in microglia, astrocytes, circulating cytokine levels, infiltration of macrophages along with changes in the transcription of genes associated with the control of inflammation, in HD (Ben Haim et al., 2015a; Crotti and Glass, 2015; Ransohoff, 2016a).

Here, we will overview the astrocytic and microglial neuroinflammatory changes in HD presented in the literature. Whether this neuroinflammatory response is protective or damaging along with its role in controlling pathways leading to cell death is still not well understood (Ellrichmann et al., 2013; Crotti and Glass, 2015). However, considerable recent research is focused on understanding the role neuroinflammation plays in the progression of the disease.

## Huntington’s Disease Pathology

HD is an autosomal dominant, genetic disease of the brain. Gene mapping studies carried out by Gusella et al. (1983) identified the genetic origin of HD as a mutation on the short arm of chromosome 4. This mutation, at locus 4p16.3, in the IT15 gene, was discovered to be an expansion of a CAG repeat in the huntingtin (HTT) gene resulting in an expanded polyglutamine tract (The Huntington’s Disease Collaborative Research Group, 1993). A CAG repeat length of up to 35 repeats generally does not result in the onset of HD (Reiner et al., 2011; Dayalu and Albin, 2015). However, individuals with repeat numbers from 36 up should be viewed at as at risk of developing HD (Ha et al., 2012; Nance, 2017). CAG repeat lengths of greater than 40 are associated with a definite onset of HD within a normal lifespan (Reiner et al., 2011). Strikingly, an inverse correlation between the age of onset and CAG repeat length has been found through correlative studies (Andrew et al., 1993). This is thought to account for around 50–70% of the variation in the age of onset (Andrew et al., 1993; Myers, 2004; Ross and Tabrizi, 2011; Dayalu and Albin, 2015).

The diffuse degeneration of the striatum has become one of the hallmarks of HD progression (Vonsattel et al., 1985; Vonsattel, 2008). Degeneration in the striatum (caudate nucleus and putamen) has been found to move in the caudo-rostral, dorso-ventral and medio-lateral directions (Vonsattel et al., 1985). Brain weight is reduced in later stages of the disease and this is consistent with the reported heterogeneous cortical degeneration. Though initially described as degeneration of the striatum, widespread cortical atrophy has been reported in the HD brain. The degeneration of these brain regions has been linked to the presentation of clinical symptoms (Thu et al., 2010; Kim et al., 2014; Nana et al., 2014; Mehrabi et al., 2016; Singh-Bains et al., 2019).

Choreatic movements were first described in patients by George Huntington (Huntington, 2003). Since then a multitude of symptoms including movement-, cognitive-, mood- and psychiatric disorders have been identified in patients. As the disease progresses, the symptoms that patients present with change and vary between cases (Novak and Tabrizi, 2011). Some patients mainly exhibit motor dysfunction at clinical onset, and few if any mood symptoms. While others have severe mood and/or cognitive dysfunction at the clinical onset, but no or few movement changes. Others experience concomitant motor, mood and cognitive symptoms at onset. This clinical variability is demonstrated well in monozygotic twins with identical HD genes but marked differences in their behavioral symptoms (Georgiou et al., 1999).

The most common motor behavior symptom of HD is chorea, these unwanted rapid, short-lasting movements can be observed in all muscles of the trunk, face, and extremities. All patients also show mild or more severe parkinsonian symptoms, slowness in starting movement (akinesis), slowness in executing a movement (bradykinesia), showing fewer spontaneous automatic movements, and decrease in all motor activities (hypokinesia). The unwanted movements and increased muscle tone can result in dystonia, slowly twisting and turning movements. Tics, mainly occurring in the face and the arms are sometimes present but the patients are usually aware of these movements and some can learn to suppress them. Every patient has a specific pattern of motor behavior that is genetically and culturally determined (Shannon, 2011).

HD has been associated with pathological changes in multiple brain regions and the manifestation of cognitive symptoms effect a wide variety of skills. Declines in cognitive processing speed, executive function, working memory, visuomotor control and time production have been reported (Paulsen et al., 2017). Difficulties to maintain attention also lead to a reduction in memory function and in later stages of the disease a complete clinical picture of dementia develops (Nance, 2017). The most common psychiatric symptoms, with prevalence of 33%-76%, include depressed mood, anxiety, irritability, and apathy. Obsessive-compulsive symptoms and psychosis occur less often, with prevalence of 10%–52% and 3%–11%, respectively (van Duijn et al., 2007). Loss of body weight, sleep disturbances and autonomic disturbances that include hyperhidrosis, heat and cold intolerance, sialorrhoea, micturition and swallowing difficulties, and sexual dysfunction are secondary signs of HD (Aziz et al., 2010).

## Microglia

**Figure 1:**
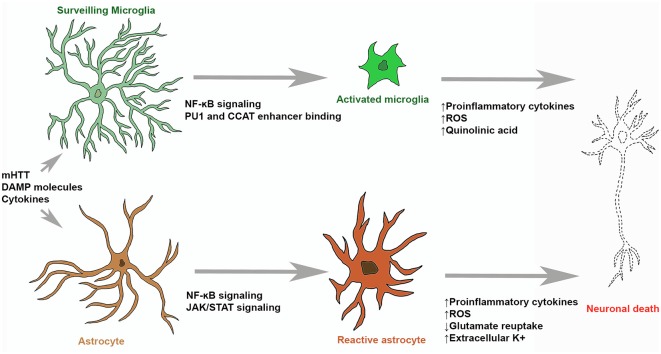
Microglial and astrocytic contribution to neuronal death in Huntington’s disease (HD). Surveilling microglia are activated by stimulating molecules through NF-κB signaling, upregulation of PU1 and CCAT binding. Activated microglia and reactive astrocytes produce reactive oxygen species (ROS) and neurotoxic molecules (such as quinolinic acid) which can induce molecular processes leading to neuronal death. Stimulatory molecules also induce reactive astrogliosis that leads to the upregulation of pro-inflammatory cytokine production, glutamate excitotoxicity and hyperexcitability of neurons.

Making up 5%–10% of the cells in the brain, microglia have long been considered the resident immune cells of the brain (Frost and Schafer, 2016). When no inflammatory stimulus is present, microglia are in a surveilling state. Following an inflammatory stimulus, microglia become activated in order to respond to the stimulus and take part in an inflammatory response (Figure 1). Changes in morphology and transcriptional activation take place in the transition of microglia from surveilling to activated (Boche et al., 2013; Crotti and Glass, 2015). Surveilling microglia exhibit a ramified morphology. These microglia show more extensive branching and processes when in a surveilling state (Glenn et al., 1992). While in the surveilling state, microglial cells survey the area around their cell bodies, by dynamic reorganization of the microglial processes, to ensure the maintenance of homeostasis. This was demonstrated through an elegant set of experiments by Nimmerjahn et al. (2005) using *in vivo* two-photon imaging of the neocortex, they showed that microglial processes displayed remarkable motility and were able to cyclically form and withdraw. Microglia further showed filopodia like protrusions along their processes. Microglia maintain a surveilling state due to the presence of contact receptor-ligand interactions, such as neuronal CD200 and microglial CD200 receptors, molecules released from neurons, such as CX3CL1 (also known as fractalkine) that act on microglial CX3CL1 receptors, and lipid mediators released from microglia, such as endocannabinoids (Hanisch and Kettenmann, 2007; Benarroch, 2013).

In the surveilling state, microglia have multiple roles in homeostasis. One such role is the maintenance of synapses and synaptic plasticity through facilitation of synaptic maturation, pruning and elimination (Tremblay and Majewska, 2011; Schafer and Stevens, 2015). This is achieved through the interactions between microglial processes, synaptic clefts, spines, termini and astrocytic processes which allow microglia to alter neural activity and structural environmental components. Microglia also facilitate the role of growth and development of surrounding neural networks by secreting neurotrophic factors, such as brain-derived neurotrophic factor (BDNF), nerve growth factor (NGF) and insulin-like growth factor (IGF-1; Nayak et al., 2014). There is significant evidence to suggest a role of microglia in promoting neurogenesis by phagocytosing apoptotic neural progenitor cells, facilitating the migration and differentiation of neural progenitor cells and secreting soluble factors which promote neurogenesis (Lira-Diaz and Gonzalez-Perez, 2016). Finally, upon detecting inflammatory stimuli, surveilling microglia can become activated and are a major part of the neuroinflammatory response (Crotti and Glass, 2015; Hickman et al., 2018). Following detection of inflammatory stimuli, such as damage associated molecular proteins (DAMPs) molecules and cytokines, microglia undergo morphological changes entering into an activated state (Boche et al., 2013). Activated microglia have a phagocytic role and are motile. Along with an active role in neuroinflammation, activated microglia also have been shown to have a role in synaptic plasticity and neuronal growth and survival (Lull and Block, 2010).

Activated microglia can adopt different states, commonly this polarization has been categorized as M1 and M2 states, and microglial cells can alternate between the two states. Recently the use of M1/M2 terminology to categorize microglial activation has been questioned (Ransohoff, 2016b) however, we will introduce the concept before discussing why this terminology is outdated in the light of recent research. M1 microglia are suggested to have a more “classic” role in the inflammatory response and are thought to be the major initiators of both innate and adaptive immunity in the brain (Nakagawa and Chiba, 2014). These cells have a phagocytic function and will release cytotoxic factors such as nitric oxide (NO), reactive oxygen species (ROS) and quinolinic acid to confer toxicity to invading pathogens (Lull and Block, 2010; Benarroch, 2013; Nakagawa and Chiba, 2014). Promotion of nuclear factor of kappa light polypeptide gene enhancer in B-cells (NF-κB) regulated transcription further results in the release of pro-inflammatory cytokines such as tumor necrosis factor alpha (TNF-α), interleukin 1 β (IL-1β) and interleukin 6 (IL-6; Lull and Block, 2010; Benarroch, 2013). These, pro-inflammatory cytokines enhance and stimulate the inflammatory response. Activated M1 microglia also show an upregulation in surface proteins which confer a role in an innate inflammatory response, such as MHC proteins which are critical in antigen presentation to T-cells (Lull and Block, 2010; Benarroch, 2013).

M2 microglia also carry out phagocytosis but contrary to the role of M1 microglia, M2 microglia exhibit an anti-inflammatory role (Nakagawa and Chiba, 2014). This is through the release of anti-inflammatory mediators such as interleukin 4 (IL-4), interleukin 13 (IL-13), IL-10 and transforming growth factor beta (TGF-β) to suppress inflammatory responses (Nakagawa and Chiba, 2014; Tang and Le, 2016). Anti-inflammatory cytokines inhibit initial phases of the immune response. Given the opposing roles of M1 and M2 microglia, it has been postulated that polarization of microglia to M1 or M2 states controls either promotion or resolution of inflammation in the brain (Nakagawa and Chiba, 2014). M2 microglia also have a role in tissue repair following the detection of anti-inflammatory cytokines, such as IL-13 and IL-4, from T helper cells and signals from apoptotic cells (Benarroch, 2013; Tang and Le, 2016).

However, *ex vivo* genome-wide expression profiling of microglia from different disease model mice failed to support the M1/M2 polarization theory (Chiu et al., 2013; Morganti et al., 2016; Wes et al., 2016). While polarization along the M1/M2 axis was not found, a consistent expression pattern profile has been observed specific for healthy and disease states in mice (Chiu et al., 2013; Bennett et al., 2016; Wes et al., 2016). Furthermore, microglia demonstrate a dynamic expression phenotype influenced by the local environment and stimuli but there is no evidence that this response promotes repair (Ransohoff, 2016b). The M1/M2 terminology in the light of findings from several recent studies has to be revised and based on transcriptomic and proteomic profiles of microglia obtained *ex vivo* from experimental subjects of healthy and different disease states, with trauma, infection, during development and aging (Bennett et al., 2016; Ransohoff, 2016b; Wes et al., 2016).

In the aged brain, microglia have also been observed to adopt an increased inflammatory profile (Norden and Godbout, 2013). This is a phenomenon referred to as microglial priming and refers to the upregulation of proteins such as major histocompatibility complex class II (MHCII) molecules, integrins and toll-like receptors which are activated in an inflammatory response (Norden and Godbout, 2013). This may contribute to the chronic inflammation seen in several neurodegenerative diseases including AD, PD and HD.

## Microglial Contribution to Huntington’s Disease

### Microglial Pathology

Microglia are primary mediators of neuroinflammation (Ransohoff and Perry, 2009; Ellrichmann et al., 2013; Crotti and Glass, 2015; Ransohoff, 2016a; Hickman et al., 2018). Changes in morphology and gene expression in microglia may underpin neuronal death in HD (Figure 1). When an insult to the brain occurs, surveilling microglia become activated and initiate innate immunity in the CNS. With continued activation of microglia, prolonged production of inflammatory mediators by microglia results in chronic inflammation and implicated in further tissue damage (Ellrichmann et al., 2013). Atrophy of the striatum (caudate nucleus, putamen and globus pallidus) is the neuropathological hallmark of HD (Vonsattel et al., 1985). Immunohistochemical experiments in human HD brain tissue identified the presence of reactive microglia in the neostriatum, cortex and globus pallidus which were absent in control brain tissue (Sapp et al., 2001; Vonsattel et al., 1985, 2011). A study also noted that the number of activated microglia in the striatum and cortex showed a direct correlation with degree of neuronal loss and that microglia were closely associated with pyramidal neurons, suggesting that neuroinflammatory changes might be induced by the degenerating neurons (Sapp et al., 2001). In fact, infiltration of peripheral inflammatory cells is not an usual feature of HD (Sapp et al., 2001; Vonsattel et al., 1985). A study by Pavese et al. (2006) used Positron Emission Tomography (PET) imaging to identify markers of activated microglia and striatal GABAergic neurons in HD patients at varying stages of disease progression showed increased microglial activation with greater disease severity and striatal neuron loss. Interestingly, they also identified an increase in microglial activation in the anterior cingulate and prefrontal cortices (Pavese et al., 2006; Tai et al., 2007a,b). Microglia activation was also identified in PET studies undertaken in presymptomatic HD gene carriers. These studies identified microglial activation as an early change in HD, prior to symptom onset (Tai et al., 2007b; Politis et al., 2015). Increased microglial activation was also detected in the striatum of presymptomatic R6/2 mice, a mouse model of HD expressing human mHTT exon 1 containing 150 CAG repeats (Simmons et al., 2007).

As mentioned above influx of peripheral immune cells such as lymphocytes and neutrophils has not been found in the HD brain and neuroinflammation is most likely sustained by the interactions of microglia, macroglia, neurons and inflammatory mediators released by these and other cells in the brain parenchyma or possibly infiltrated from periphery (Sapp et al., 2001; Silvestroni et al., 2009; Vonsattel et al., 1985). The literature suggests an upregulation of activated microglia in a number of human and animal HD studies and also reporting increases in cytokines secreted by microglia (Björkqvist et al., 2008; Yang et al., 2017). Peripheral inflammatory response has been well described in HD, key cytokines of the innate immune system are upregulated both centrally and peripherally. C-reactive protein and cytokine profile were altered in human plasma and mouse serum samples from three different mouse models (Stoy et al., 2005; Björkqvist et al., 2008; Sánchez-López et al., 2012). Elevations in IGF-β, IL-1β, IL-6, IL-8, IL-10, TNF-α, TGF-β, CCL2 and matrix metallopeptidase 9 (MMP-9) were measured in HD patient plasma, CSF and post-mortem brain tissue using ELISA and quantitative real time polymerase chain reaction (qRT-PCR; Björkqvist et al., 2008; Silvestroni et al., 2009; Chang et al., 2015; Politis et al., 2015). Some of these inflammatory mediators such as IL-1β and TNF-α showed increased levels only in the striatum but others, IL-6, IL-8 and MMP-9 were also upregulated in cortical regions and the cerebellum. These findings suggest that in contrast to other neurodegenerative diseases, such as AD and PD with a more generalized neuroinflammatory profile and a wide range of inflammatory mediators, the HD brain is characterized with a very different neuroinflammatory characteristics (Möller, 2010). In HD, cytokines such as IL-4 and IL-10 are increased at later stages of the disease, but normal Ig levels throughout the disease course suggest that there is no generalized activation of the adaptive immune response (Björkqvist et al., 2008). IL-6 and IL-8 production are triggered by NF-κB activation, while IL-4 and IL-10 act to downregulate NF-κB (Khoshnan et al., 2004; Björkqvist et al., 2008). This might suggest an adaptive response to chronic immune activation.

The correlation between plasma and CSF levels of cytokines such as IL-6 and IL-8 suggests that the central and peripheral immune activation in HD are linked. There is evidence that these cytokines do not cross the healthy blood-brain barrier and they are rapidly broken down after release but passage between the CNS and blood still remains a possibility (Steensberg et al., 2006; Pan and Kastin, 2007; Björkqvist et al., 2008). While microglia are considered as one of the main sources of cytokine production in the HD brain, their release could also be caused by monocytes, macrophages, astrocytes and other cell types such as endothelial cells and pericytes which are components of the neurovascular unit (Björkqvist et al., 2008; Govindpani et al., 2019). The role of upregulated mHTT in this process has been extensively investigated.

### mHTT Accumulation and Microglial Activation

mHTT accumulation in neurons also has been linked to the activation of microglia. In primary neuronal cultures and cortico-striatal brain slices, from rat and mouse brain tissue, elevated numbers of microglia with an activated phenotype were observed in concurrence with the expression of mHTT in neurons and these microglia were positioned along irregular neurites (Kraft et al., 2012). The presence of mHTT inclusions in microglia have also been identified (Jansen et al., 2017). The cell autonomous expression of mHTT has also been implicated in the activation of microglia (Crotti et al., 2014; Yang et al., 2017). In genome wide studies, Crotti et al. (2014) found that an increase in the expression and transcriptional activity of PU.1 and CCAT/enhancer-binding protein in mHTT expressing microglia when compared to microglia not expressing the mHTT. The authors postulate that this leads to the enhanced transcription of basal pro-inflammatory gene expression and, in turn, increased levels of pro-inflammatory cytokines can perpetuate further inflammation and tissue damage. Furthermore, astrocytes isolated from R6/2 mice observed to react hyperactively to stimulation when compared to those isolated from wildtype mice, with the authors of this study postulating this was due to mHTT expression in microglia (Björkqvist et al., 2008). A recent study demonstrated that microglia expressing mHTT show significant elevations in NF-κB when stimulated with IL-6 but not when stimulated with LPS. This suggests that mHTT may alter immune responses in microglia in a stimuli dependent manner (Donley et al., 2019). However, the degree of the effect these microglial changes have on pathology of HD has come under scrutiny in a recent study. In this study depletion of mHTT in microglia of BACHD mice showed no significant rescue of behavioral performance, brain weight or striatal and cortical volume. Conversely depleting mHTT in all other cells except microglia resulted in rescue of behavioral performance and neuropathology (Petkau et al., 2019). However, while the presence of microglial changes due to the presence of mHTT may not be sufficient to induce the HD phenotype, it may contribute to pathology in conjunction with further changes in the brain. A future area of research could be to carry out similar studies on different animal models to corroborate these findings.

### Major Signaling Pathways Implicated in Microglial Activation

A number of key signaling pathways have been implicated in the activation of microglia in HD. These include NF-κB signaling, the kyurenine pathway and the cannabinoid receptor pathway. As discussed above, the NF-κB signaling has been elucidated in the literature as a key pathway through which mHTT induces the activation of microglia in HD (Khoshnan et al., 2004; Khoshnan and Patterson, 2011). mHTT interacts with the IκB kinase (IKK) γ subunit of the IKK complex. This promotes the assembly and activation of the IKK complex which contains the IKKα and IKKβ subunits. The IKKβ kinase phosphorylates IκBα, causing the liberation of NFκB to promote the gene expression of pro-inflammatory cytokines (Häcker and Karin, 2006; Khoshnan and Patterson, 2011). Khoshnan et al. (2004) demonstrated through immunohistochemistry, immunoprecipitation, western blotting and GST pulldown assays that mHTT interacts with IKKγ in a PC12 cell culture and that the IKK complex showed increased activity. This has also been observed in the striatum and cortex of R6/2 mice expressing mHTT (Khoshnan et al., 2004). Träger et al. (2014) corroborated that mHTT interacts with the IKK complex to allow signaling *via* the NF-κB pathway in peripheral blood mononuclear cells from HD patients. Lowering mHTT *via* the use of targeted siRNA alleviated excessive NF-κB signaling with a reduction in the levels of pro-inflammatory cytokines (Träger et al., 2014). Few recent studies have reported the interaction of mHTT and NF-κB in microglia in particular, but the presence of such interaction in myeloid cells and monocytes suggests that such a pathway may underlie the activation of microglia in HD.

A growing area of research in the role of microglia in HD focuses on the kynurenine pathway. This pathway for L-tryptophan metabolism produces several metabolites with neuroactive properties (Schwarcz et al., 2010). Tryptophan is metabolized to the neurotoxic quinolinic acid (QUIN) and 3-hydroxykynurenine (3HK) by the enzyme kyurenine 3-monooxygenase (KMO). QUIN is postulated to have a role in neurotoxicity as it is a selective agonist of N-Methyl-D-aspartic acid (NMDA) receptors while 3HK has a role in potentiating QUIN. Genome wide screening in yeast found the genes *BNA4* and *BNA1* coding for KMO and 3-hydroxyanthranilate 3, 4 dioxygenase respectively, regulated mHTT toxicity through its role in the production of QUIN and 3HK (Giorgini et al., 2005). In low-grade human HD tissue, elevations in 3HK and QUIN have been reported in the neostriatum and neocortex however these levels remained unchanged or declined with further progression of the disease. Therefore, it was postulated that the neurotoxic contribution kyurenine pathway metabolites may be involved in the early stages of HD pathophysiology (Guidetti et al., 2004). 3HK levels were elevated in the striatum and cortex of R6/2 mice and in primary microglial cultures from R6/2 mice and KMO activity was elevated in R6/2 mice as well (Giorgini et al., 2008). This increase of neurotoxic metabolites of the kynurenine pathway can be reduced in R6/2 mice by treatment with histone deacetylase inhibitors and these results might suggest the use of these agents as potential therapeutics to treat HD (Giorgini et al., 2008).

Altered activation of the cannabinoid pathway has also been observed in HD. Signaling through CB2 receptors have been shown to reduce immune activation in the brain. Real time PCR experiments revealed increased CB2 transcript levels in R6/2 mice, and mice with a genetic deletion of CB2 showed increased microglial activation and worse behavioral performance than standard R6/2 mice (Palazuelos et al., 2009). Western blotting experiments further showed an elevated expression of CB2 receptors in the caudate and putamen of HD patients when compared to control tissue (Palazuelos et al., 2009). Further immunohistochemistry experiments revealed expression of CB2 receptors on microglia striatum and the caudate-putamen of HD patients and R6/2 mice, but not on astrocytes. CB2 was also found expressed in microglia using immunohistochemistry in a malonate lesion rat model of HD (Sagredo et al., 2009). Dowie et al. (2014) however, reported that in human post mortem tissue there was an absence in CB2 receptors in microglia along with astrocytes using immunohistochemistry. This discrepancy may be due to the markers for microglia used. Dowie et al. (2014) used Iba1 while Palazuelos et al. (2009) used CD-68. Along with microglia, CD-68 is also present in monocytes which might have been stained in this study as well. A further study determined that CB2 ablation in BACHD mice resulted in poorer performance in the balance beam behavioral task (Bouchard et al., 2012). However, this study concluded that suppression of neuroinflammation is likely through CB2 signaling in peripheral immune cells rather than resident microglia in the brain. These findings highlight a weakness in current mouse models at recapitulating HD, wherein mouse models, it appears that microglia are responsible for inflammatory suppression through CB2 signaling pathways while this does not appear to be the case in the humans.

## Astrocytes

Astrocytes are one of the most prevalent glial cell types in the mammalian brain (von Bartheld et al., 2016). They are characteristic star-shaped cells with many processes that envelop synapses made by neurons. Astrocytes have specialized processes called astrocyte end-feet that extend from the astrocyte cell body and attach to the basement membrane that surrounds the endothelial cells and pericytes on the brain vasculature. Over the last few decades increasing evidence suggests that besides their housekeeping function astrocytes carry out a number of roles in maintenance and control of healthy brain function (Verkhratsky and Nedergaard, 2018). They are involved in synaptic function by regulating synapse formation and maturation, neurotransmitter homeostasis and release of gliotransmitters; they regulate pH, water and ion homeostasis; they form an integral part of the blood-brain barrier and are involved in regulation of neurovascular coupling, vascular tone and blood flow (Simard and Nedergaard, 2004; Benarroch, 2005; Blackburn et al., 2009; Sofroniew and Vinters, 2010; MacVicar and Newman, 2015; Verkhratsky and Nedergaard, 2018; Govindpani et al., 2019). Astrocytes form broad cellular networks connected through gap junctions, which allow intercellular diffusion of ions, second messengers and small molecules. Astrocytic intercellular diffusion has been reported for cyclic AMP, inositol-1,4,5-trisphosphate (InsP3), Ca^2+^, glutamate, adenosine triphosphate (ATP) and energy metabolites (glucose, glucose- 6-phosphate and lactate; Nagy et al., 1999; Tabernero et al., 2006). Neurovascular coupling, the local perfusion that occurs in response to changes in neuronal activity, is primarily mediated at the level of the neurovascular unit (Muoio et al., 2014). Astrocytes as integral parts of the neurovascular unit are key regulators of neurovascular coupling and help to meet dynamic energy requirements in the brain. As mentioned above astrocytes and other components of the NVU are functionally linked *via* gap junctions, adhesion molecules and the local release of vasoactive agents and neuromodulators. The release of these molecules in particular by interneurons and astrocytes is critical for the rapid and precise modulation of blood flow as well as vascular cell function and homeostasis in response to local metabolic demand (Muoio et al., 2014; Nuriya and Hirase, 2016; Mishra, 2017).

Astrocytic processes lie in close contact with neuronal somata, bundles of axonal internodes and synapses. Astrocytes mediate synaptic function through controlling the levels of various neurotransmitters at the synapse (Blackburn et al., 2009; Chung et al., 2015; Sofroniew and Vinters, 2010). By controlling the levels of γ-aminobutyric acid (GABA) production through provision of glutamine to neurons, and GABA clearance, affecting the time that GABA is left at the synaptic cleft, astrocytes regulate the inhibition of post-synaptic neurons (Blackburn et al., 2009; Schousboe et al., 2013; Sofroniew and Vinters, 2010). Glutamine is also a precursor to the synthesis of glutamate and glutamatergic neurons are supplied glutamine by astrocytes after astrocytic uptake of synaptic glutamate and its metabolism (Bélanger and Magistretti, 2009). Cycling of glutamine to neurons ensures that depletion of glutamate at synapses does not occur and neurotransmission can resume (Bélanger and Magistretti, 2009). The uptake of glutamate from the synapse by astrocytes is particularly important as prolonged exposure to excess glutamate can result in glutamate-mediated excitotoxicity (Bélanger and Magistretti, 2009).

Astrocytes have a role in ion homeostasis at the synapse. K^+^, Na^+^, HCO^3-^, Cl^−^ and H^+^ ion levels are all regulated by astrocytes to ensure neuronal cell survival and continued efficient neurotransmission (Simard and Nedergaard, 2004). Along with mediating homeostasis within the synapse, astrocytes also exhibit an active role in neuronal signaling (Simard and Nedergaard, 2004; Blackburn et al., 2009; Sofroniew and Vinters, 2010). The pre-synaptic neuron, post-synaptic neuron and astroglial cell make up the tripartite synapse. The release of glial transmitters such as glutamate, GABA, ATP and D-serine impact excitation and inhibition of the CNS. Water homeostasis is achieved through the presence of aquaporins on astrocytes, such as AQP4, in the plasma membrane. These are especially prevalent in astrocytic processes surrounding blood vessels and play a critical role in fluid homeostasis (Benarroch, 2005).

Astrocytic end-feet come into close contact with the endothelial cells of the vasculature and form part of the blood-brain barrier (Abbott et al., 2006). Further evidence also suggests that astrocytes are able to secrete angiogenic factors to propagate the formation of capillaries in the brain (Blackburn et al., 2009; Sofroniew and Vinters, 2010). Takano et al. (2006) identified that astrocytes have a role in blood vessel diameter regulation with vasodilation noted in blood vessels ensheathed by astrocytic end-feet.

Following an insult to the brain, astrocytes undergo a process known as “reactive astrogliosis” (Figure 1; Zamanian et al., 2012). Here, astrocytes undergo significant morphological changes along with significant gene expression alterations (Wilhelmsson et al., 2006; Sofroniew, 2009), they become hypertrophic and show enhanced GFAP expression (Ben Haim et al., 2015a). This process has been found to be both protective and harmful. Of particular note in the context of inflammation is that reactive astrocytes have been shown to produce chemokines such as chemokine C-C motif ligand 2 (CCL2) and C-X-C motif ligand 1 (CXCL1), pro-inflammatory cytokines such as interleukin 12 (IL-12) and TNF-α, and anti-inflammatory cytokines such as interleukin 10 (IL-10) and TGF-β (Cekanaviciute and Buckwalter, 2016). Reactive astrogliosis is a gradual continuum of changes that occur in context-dependent manners regulated by specific signaling events. Astrocytes, like microglia, are actively maintained in a resting state. The exact molecular triggers of astrocyte and microglia reactivity during initial stages of neurodegenerative disorders, before significant neuronal loss are unknown but it was suggested that they might detect altered neurotransmission, release of stress signals and abnormally folded proteins (Ben Haim et al., 2015a). Furthermore, cytokines, growth factors and purines can activate these cells and intracellular signalling cascades that will lead to their transition into reactive states (Buffo et al., 2010; Ben Haim et al., 2015a).

Recent findings also suggest that reactive astrocytosis in certain context being an adaptive beneficial response. Astrocytosis could lead to increased neuroprotection and trophic support of insult-stressed neurons; isolation of the damaged area from the rest of the CNS tissue, reconstruction of the blood brain barrier and the facilitation of remodeling of brain circuits following an insult (Buffo et al., 2010; Pekny et al., 2016). Reactive astrocytes can acquire properties of stem cells and support the remodeling of neuronal circuits, and removal of these cells can increase tissue damage and neuronal death (Robel et al., 2011; Sofroniew and Vinters, 2010). Astrocytes also produce high levels of antioxidants for neurons, including ascorbic acid, glutathione and its precursors (Allaman et al., 2011). However, reactive astrocytes can produce decreased levels of antioxidants and they also release more ROS and NOS in neurodegenerative disorders such as AD and HD (Ben Haim et al., 2015a). Reactive astrocytes also phagocytose debris and have a role in the repair of damaged tissue (Sofroniew and Vinters, 2010). Recent research suggests the existence of two subtypes of reactive astrocytes. These are the proinflammatory, neurotoxic A1 type and the anti-inflammatory, neuroprotective A2 type cells (Liddelow and Barres, 2017; Sofroniew, 2009). Though further subtypes have not been identified in the literature, it is hypothesized that astrocyte populations might exist in a continuum between A1 and A2 polarized states or may even exist in a number of separate activation states (Liddelow and Barres, 2017). The fate of astrocytes to either of these subtypes is dependent on the insult to the brain that occurs and cell signaling pathways that are triggered in these insults (Liddelow and Barres, 2017; Sofroniew, 2009). The “detrimental” activated astrocytes are speculated to be resultant of inflammatory stimulus and show upregulation of genes which are linked to synapse destruction leading to neuronal loss (Zamanian et al., 2012). These astrocytes are postulated to be induced through NF-κB signaling and are reported present in a number of neurodegenerative diseases including AD, PD, ALS, Multiple Sclerosis (MS) and HD (Li et al., 2019; Liddelow and Barres, 2017; Liddelow et al., 2017). The “helpful” reactive astrocytes induced by an ischemic event show the upregulation of neurotrophic factors, cytokines and thrombospondins and are postulated to stimulate synapse development and neuronal survival (Zamanian et al., 2012). In summary, reactive astrogliosis is a finely regulated continuum of molecular, cellular, and functional changes and these changes can exert both beneficial and detrimental effects in a context-dependent manner determined by specific molecular signaling cascades.

## Astrocytic Contribution to Huntington’s Disease

### Astrocyte Pathology

Previous literature suggests the contribution of astrocytes to neuron death and other hallmarks of HD pathology (Figure 1). In postmortem HD brains an increased number of astrocytes has been observed. While microglial density and activation were detected prior to symptom onset and neuropathological changes, no reactive astrocytes were found in grade 0 HD brains, reactive astrocytosis is observed only after neurodegeneration (Vonsattel et al., 1985, 2011). Importantly the levels of both microglial activation and reactive astrocytosis are correlated with disease severity. In the HD striatum, the increased number of GFAP positive reactive astrocytes is correlated with the gradient of striatal neurodegeneration (Vonsattel et al., 1985). However, this is not the case in the cortex, no astrocytosis is seen in the HD cortex in regions of neuronal loss and microglia activation (Zalneraitis et al., 1981; Sotrel et al., 1991). A prominent astrocytosis has also been detected in HD animal models (Lin et al., 2001; Gu et al., 2005, 2007; Faideau et al., 2010). Besides neuronal death reactive astrocytosis in HD might also contribute to pericyte death along the cerebral blood vessels and further accelerate progression of the disease (Hsiao et al., 2015).

There is growing evidence for the heterogeneity among reactive astrocytes across different brain regions, but also locally within the same region as regards astrocyte proliferation, morphology and gene expression. Single-cell level analysis shows that reactive astrocytes can exhibit different expression levels of chemokines or cytokines, signaling molecules and transcription factors (Herrmann et al., 2008; Garcia et al., 2010; Hamby et al., 2012). Despite this heterogeneity large-scale gene expression studies show that inflammatory mediators can drive astrocyte transcriptome profiles towards pro-inflammatory phenotypes (Hamby et al., 2012; Zamanian et al., 2012) that may be beneficial in microbial infection but can be detrimental in neurodegenerative disease (Sofroniew, 2014).

#### mHTT Accumulation and Astrocyte Activation

Recent literature also suggests mHTT is expressed and aggregates in astrocytes, and contributes to neuronal excitotoxicity *via* downregulation of glutamate transporters EAAT2/GLT1, resulting in impaired glutamate uptake (Arzberger et al., 1997; Liévens et al., 2001; Shin et al., 2005; Faideau et al., 2010; Estrada-Sánchez and Rebec, 2012; Ellrichmann et al., 2013; Khakh et al., 2017). This loss of GLT1 expression has been noted as one of the earliest signs of astrocytic dysfunction in HD (Khakh et al., 2017). In a further study, R6/2 mice were injected with ceftriaxone, an antibiotic known to raise the expression of GLT1. This study found that ceftriaxone injected R6/2 mice showed enhanced performance in behavioral tests in comparison with vehicle-injected controls (Miller et al., 2008). This study confirmed that mice injected with ceftriaxone showed greater expression of GLT1, demonstrated through western blotting and immunohistochemistry. Furthermore, microdialysis data showed a decrease in extracellular glutamate levels in the striatum of ceftriaxone injected mice when compared with vehicle control mice. It is interesting to note that a reduction in glutamate uptake in HD models can occur independently of changes in GLT-1 protein levels suggesting that the functionality of these transporters is also reduced (Estrada-Sánchez and Rebec, 2012).

Using a mouse genetic approach by breading the conditional mHTT-expressing BACHD mouse model with GFAP-CreERT2-tam mice produced offspring with significantly reduced expression of mHTT protein in the striatum and cortex. The BACHD/GFAP-CreERT2-tam mice showed better performance in the rotarod and open field tests when compared to standard BACHD mice (Wood et al., 2019). These mice also showed increased expression of postsynaptic density protein 95 (PSD-95), α actinin2 and aB-crystallin expression compared to standard BACHD mice, along with improvements in the electrophysiological phenotype (Wood et al., 2019).

Astrocytes with nuclear mHTT inclusions showed decreased expression of Kir4.1 channel, leading to impaired K^+^ homeostasis in HD (Tong et al., 2014). Kir4.1 channels allow the influx of K^+^, thereby maintaining membrane conductance in astrocytes. Recent literature suggests that, in HD, there is a loss in the expression of these K^+^ channels, in turn resulting in decreased membrane conductance in astrocytes. Kir4.1 channels were found to be reduced in striatal tissue of R6/2 and Q175 mice and significant proportion of striatal astrocytes that presented nuclear mHTT inclusions showed reduced Kir4.1 channel expression (Tong et al., 2014). It is postulated that a reduction in Kir4.1 channels results in an increase in extracellular K^+^ levels which in turn results in medium spiny neurons being 3–13 mV more depolarized in HD mouse models than in wild-type mice (Tong et al., 2014). This increased depolarization may further underlie the hyperexcitability of the striatal medium spiny neurons in HD.

mHtt expression in astrocytes, and not in neurons, is sufficient to trigger oxidative stress in neurons by diffusible factors (Boussicault et al., 2014). The release of ascorbic acid by astrocytes is altered in HD (Rebec, 2013). In the R6/2 mouse model, extracellular ascorbic acid levels are lower than in age-matched WT mice but only during behavioral activity (Rebec et al., 2002). Therefore, reactive astrocytes may not only produce fewer antioxidant molecules in the HD brain, but they might also release more pro-oxidant factors (Ben Haim et al., 2015a).

Furthermore, mHTT has been shown to reduce astrocytic release of BDNF (Wang et al., 2012; Hong et al., 2016). Interestingly, phosphorylation of mutant huntingtin at the S421 residue promotes neuroprotection in HD, by restoring huntingtin function in the transport of BDNF (Zala et al., 2004).

A recent study demonstrated the modeling of HD in *in vitro* derived monkey astrocytes and the therapeutic efficacy of astrocyte targeted RNAi, expression of small-hairpin RNA against *HTT* (shHD) ameliorated and reversed HD phenotypes in astrocytes (Cho et al., 2019). The therapeutic potential of this targeted approach has to be tested in human astrocytes.

### Major Signaling Pathways Implicated in Astrocytic Activation

In HD, the degenerating neurons, activated microglia, pericytes, endothelial cell and astrocytes release molecules that activate astrocytic intracellular signaling pathways such as the janus kinase/signal transducer and activator of transcription (JAK/STAT), NF-κB mitogen-activated protein kinase (MAPK) and calcineurin (CN). Activation of the JAK/STAT3 pathway is a universal feature of astrocyte reactivity in models of different neurodegenerative disorders (Ben Haim et al., 2015a). STAT3 accumulates in the nucleus of reactive astrocytes in the striatum of mouse and primate HD models (Ben Haim et al., 2015b). Lentiviral overexpression of suppressor of cytokine signaling 3 (SOCS3), the endogenous inhibitor of the JAK/STAT3 pathway in astrocytes *in vivo*, inhibited this pathway and prevented astrocyte reactivity but also increased the number of mHTT and did not influence neuronal death (Ben Haim et al., 2015b). N171-82Q and Hdh140 mice display behavioral abnormalities and striatal atrophy with no substantial astrocyte reactivity in endstage N171-82Q mice or before 17 months of age in Hdh140 mice. The JAK/STAT3 pathway was activated in the few reactive astrocytes of the old Hdh140 mice (Tong et al., 2014). In contrast, a significant astrocytosis is found in the brains of HD patients (Faideau et al., 2010) but this discrepancy might be related to the absence of massive neuronal death in HD transgenic models. Therefore, the JAK/STAT3 pathway seems ultimately responsible for triggering astrocyte reactivity. However, due to the crosstalk between the NF-κB and the JAK/STAT3 pathways, there is possibility that the NF-κB pathway secondarily activates the JAK/STAT3 pathway or STAT3 inhibits the NF-κB pathway and other signaling pathways are also involved in astrocyte activation (Fan et al., 2013; Ben Haim et al., 2015b). Activation of the JAK/STAT3 pathway is involved in cell proliferation, survival, and differentiation. In astrocytes the activation of the pathway increases the expression of cytoskeletal proteins, such as GFAP and vimentin, but many other genes are induced that are involved in the release of cytokines and chemokines (Burda and Sofroniew, 2014).

The NF-κB pathway have been associated with astrocyte reactivity and neuroinflammation (Kang and Hebert, 2011). Studies carried out on primary R6/2 mouse astrocyte cultures showed increased activation of NF-κB resulting in an efflux of pro-inflammatory cytokines (Hsiao et al., 2013). This study postulated that this was achieved through the enhanced activation of IkB kinase leading to prolongation of NF-κB activation, thus upregulating pro-inflammatory factors. Enhanced activation of nuclear factor NF-κB-p65 (p65) was also observed in astrocytes of patients and mice with HD (Hsiao et al., 2013). Another study that examined the abundance of IκBα, the master inhibitor of the NF-κB pathway which is degraded during pathway activation, did not show decrease in the lentivirus Htt82Q-injected mouse striatum (Ben Haim et al., 2015b). The NF-κB pathway is involved in a range of cellular processes including immune response, inflammation, cell division and apoptosis (Mattson and Meffert, 2006). Hsiao et al. (2013) showed that aberrant activation of NF-kB occurred only in HD astrocytes, and not in HD microglia or HD neurons, under basal conditions and suggest that enhanced NF-kB-mediated inflammatory response in HD astrocytes might play a crucial role in regulating the initial response of HD brains to inflammatory stimuli. Furthermore, they showed that mHTT enhanced IKK activity leading to prolonged NF-kB activation in astrocytes during inflammation. Upregulation of iNOS and increased production of inflammatory mediators (e.g., NO, TNF-α and IL-1β) by LPS and cytokines (TNF-α + IL-1β) were also observed *in vitro* in R6/2 primary astrocytes when compared with WT astrocytes. As discussed above, several studies suggest that this pathway plays a key role in microglia activation but might not be critical to initiate astrocyte reactivity, hence further studies are needed to understand the role of NF-kB in astrocytes in HD.

The MAPK pathway is activated in many cell types in patients with neurodegenerative conditions and mouse models, but there is no evidence showing that it is directly involved in the initiation of astrocyte reactivity and activation of microglia (Bachstetter et al., 2011; Ben Haim et al., 2015a). Further experiments are needed to delineate which ligands and receptors activate which components of this signaling in HD astrocytes and microglia, and determine the functionally relevant downstream genes that are regulated by the MAPK pathway.

The effects of CN on astrocyte reactivity are complex and context-dependent, CN can both trigger and prevent reactivity, particularly in AD. CN modulates transcription factors such as nuclear factor of activated T-cells (NFATs) and NF-κB (Buffo et al., 2010; Ben Haim et al., 2015a). Increase in calcineurin activity in the brain of *Hdh*Q111/Q111 HD knock-in mice can lead to the selective loss of huntingtin phosphorylation and contributing to neuronal cell death in HD. Inhibition of calcineurin by FK506 led to sustained phosphorylation of mutant huntingtin at S421 and restored BDNF transport in rat primary neuronal cultures expressing mHTT and mouse cortical neurons from *Hdh*Q111/Q111 mice (Pineda et al., 2009). Based on previous AD studies CN seems to modulate rather than induce astrocyte reactivity but more research is required to determine the role of CN in HD astrocytes (Ben Haim et al., 2015a).

### Reactive Astrocytes Confer a Neurotoxic Role in HD?

The role of reactive astrocytes in HD progression is not well understood. Some studies suggest that in HD there is an imbalance in the neuroprotective and neurotoxic roles of reactive astrocytes favoring neurotoxicity (Sofroniew, 2009). A loss of neuroprotective function is likely due to the genetic mutation causing HD which results in abnormal, dysfunctional astrocytes expressing the mHTT protein and the promotion of neuroinflammation as discussed above. Furthermore, dysfunctional astrocytes lose their ability to provide support for neurons and this might lead to the neuronal death reported in HD. Although based on these studies reactive astrocytes are generally considered detrimental for neuronal function, this is mainly due to the lack of studies focusing on their beneficial effects (Khakh et al., 2017; Liddelow and Barres, 2017). Another important point is, while a few studies suggest that reactive astrocytes might have beneficial effects and promote neuronal survival in neurodegenerative and other CNS disorders, there is a lack of HD focused research (Escartin and Bonvento, 2008; Hamby and Sofroniew, 2010). Importantly, recent studies are just the start of beginning to understand the contribution of astrocyte to HD in mouse models but their contribution to disease pathology in the human brain remains to be determined. Therefore, the analysis of human HD specimens is critical and urgently needed.

## Neuroinflammation Targeted HD Therapeutics

**Figure 2:**
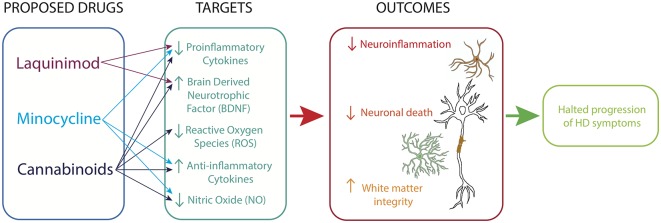
Immunomodulatory therapy for HD. Drugs acting on microglia, astrocytes and neurons reduce neuroinflammatory processes, promote neurotrophic support and sustain white matter integrity.

Currently there are no therapeutic treatments available for the cure of HD. Though the presence of chronic inflammation has been established in HD, few targeted therapies have been proposed (Figure 2). Minocycline is a second-generation tetracycline with anti-inflammatory and antiapoptotic properties (Rocha et al., 2016). A metanalysis study looking at the efficacy of minocycline in rodents found that a low dose (5 mg/kg/day) of minocycline brought about a large treatment benefit and better behavioral performance of mice (Li et al., 2013). A 2 years clinical trial of minocycline administered at a dose of 100 mg/day showed promising results with a stabilization of general neuropsychological and general motor symptoms and, a significant amelioration in psychiatric symptoms (Bonelli et al., 2004). However, it is important to note that this study had a low sample size of 11 patients and therefore these results needed to be corroborated in studies with more patients treated. A further clinical trial on 30 patients over a 6 months period found a trend toward improvement on the Unified Huntington’s Disease Rating Scale (URHDS; Thomas et al., 2004). The authors noted that lack of statistical significance may be due to the relatively short treatment period. A larger study of 87 HD patients over 18 months assessed the potential benefits of this drug treatment in a phase 3 clinical trial (clinicaltrials.gov ID: NCT00277355) and found that patient improvement did not reach a futility threshold, based on these findings further trials of minocycline 200 mg/day were not warranted (Cudkowicz, 2010).

Cannabinoids have also been seen as potential therapeutic agents due to their anti-inflammatory properties (Stella, 2009; Möller, 2010; Rocha et al., 2016). A clinical trial of cannabidiol on 15 HD patients showed no improvements in motor function over a 6 weeks period (Consroe et al., 1991). Again, this study had a low sample size, therefore caution must be taken when concluding the efficacy of cannabidiol. Similarly, a double-blind, randomized, placebo-controlled, cross-over pilot clinical trial with Sativex^®^, a botanical extract with an equimolecular combination of delta-9-tetrahydrocannabinol and cannabidiol conducted over 12 weeks on 24 HD patients reported no significant symptomatic effects (clinicaltrials.gov ID: NCT01502046; Moreno et al., 2016).

Liquinimod is an immunomodulator which acts to reduce the production of pro-inflammatory cytokines. Liquinimod treatment of YAC128 mice resulted in improved motor function and motor learning as well as a reduction in depressive-like behaviors (Garcia-Miralles et al., 2019). A clinical trial of liquinimod HD patients has been completed (LEGATO-HD, clinicaltrials.gov ID: NCT02215616). While the results are yet to be published, it has been reported that the drug failed to meet the primary endpoint of the trial (Carroll, 2018).

The failure of these clinicals trials might suggest that the effects of anti-inflammatory treatments (e.g., nonsteroidal anti-inflammatory drugs) and global immunosuppression (e.g., corticosteroids) are probably too broad, current approaches do not consider the high heterogeneity in microglia and astrocyte phenotypes (Ransohoff, 2016b; Masgrau et al., 2017; Matias et al., 2019). A better understanding of the characteristics and function of these cells in healthy and different disease states, trauma, infection, during development and aging is absolutely critical. These cells may gain gain toxic function and produce factors that are harmful to neurons and other cell types in the CNS but there is no convincing evidence of astrocyte or microglia-dependent toxicity in human patients in most neurodegenerative condition, including HD, AD and PD (Masgrau et al., 2017). This highlights the need for more studies on human materials such as primary cell lines, neural progenitor cells generated from induced pluripotent stem cells and differentiated to glial cells and biopsy-derived microglia and astrocytes. These active processes are difficult to study in the human brain but detailed anatomical studies investigating the relationship of the various types of glial cells in relation to neuronal dysfunction in the various regions of the post-mortem human brain could reveal more on the glial-neuronal interactions in HD. As there is a large heterogeneity in cell loss throughout the brain which is linked to symptomatology (Nana et al., 2014), regions of high cell loss can be compared with regions which are spared or are less affected by the disease process. These combined research studies could lead to the development of successful or improved HD therapies.

## Concluding Remarks

The current understanding of neuroinflammation in HD exposes a crucial role of astrocytes and microglia to the progression of HD. The role of these cells in the perpetuation of chronic inflammation and neuronal death highlights these cells as a promising therapeutic target for HD. There are no currently available disease-modifying treatments for HD. In recent years, RNA interference has emerged as the most promising area of therapeutic development for the disease. Currently, the development of antisense oligonucleotides to mHTT are providing a promising future therapy for HD. As discussed in this review, mHTT interacts with astrocytes and microglia to perpetuate neuroinflammation and subsequent cell death. Therefore, the clearance of mHTT using antisense oligonucleotides could possibly lead to the reduction of neuroinflammation. Furthermore, using these RNA interference therapeutics in conjunction with anti-inflammatory therapies might lead to more effective treatment of HD.

## Author Contributions

TP and AK prepared the figure. TP, HW, RF and AK wrote the article.

## Conflict of Interest

The authors declare that the research was conducted in the absence of any commercial or financial relationships that could be construed as a potential conflict of interest. The reviewer CR declared his involvement as co-editor in the Research Topic, and confirms the absence of any other collaboration.
